# Persistence of Social Norms Feedback on Postsurgery Opioid Prescribing Behavior: Secondary Analysis of a Randomized Clinical Trial

**DOI:** 10.1001/jamahealthforum.2024.5279

**Published:** 2025-01-31

**Authors:** Kyle A. Zanocco, Zachary Wagner, Louis T. Mariano, Allison Kirkegaard, Xiaowei Yan, Craig R. Fox, Noah J. Goldstein, Chad M. Brummett, Katherine E. Watkins

**Affiliations:** 1 Section of Endocrine Surgery, Division of General Surgery, Department of Surgery, David Geffen School of Medicine, University of California, Los Angeles; 2 Center for Economic and Social Research, University of Southern California, Los Angeles; 3 RAND Corporation, Arlington, Virginia; 4 RAND Corporation, Santa Monica, California; 5 Palo Alto Medical Foundation, Palo Alto, California; 6 Anderson School of Management, Department of Psychology, College of Letters and Sciences, and David Geffen School of Medicine, University of California, Los Angeles; 7 Division of Pain Medicine, Department of Anesthesiology, University of Michigan Medical School, Ann Arbor

## Abstract

This secondary analysis of a randomized clinical trial evaluates the persistence of prescribing guideline-recommended postoperative opioids 1 year after intervention cessation.

## Introduction

Prescribing excess opioids to surgical patients increases the risk of chronic opioid use and diversion of unused opioids.^1,2^ In a recent cluster randomized clinical trial (RCT), social norms–based feedback to surgeons prescribing postoperative opioids above guideline-recommended quantities showed significantly reduced guideline-discordant prescribing behavior during the intervention period.^3^ This study describes the persistence of effects 1 year after stopping the interventions.

## Methods

This cluster RCT was conducted among general, orthopedic, and obstetric and gynecologic surgical specialties at 19 hospitals in California between October 19, 2021, and October 18, 2022.^3^ This secondary analysis and waiver of participant informed consent were approved by the Sutter Health Institutional Review Board. The trial protocol is provided in Supplement 1. This study followed CONSORT reporting guidelines.

Surgeons were randomized to receive feedback on opioid prescribing relative to institutionally endorsed guidelines (guidelines intervention) or peer surgeons (peer comparison intervention) or to a control group (no intervention). Surgeons in the intervention groups received monthly email feedback after operating on patients discharged with opioid prescriptions exceeding procedure-specific guidelines. The primary outcome was the share of discharge opioid prescriptions for which total quantities exceeded guideline recommendations. As a prespecified secondary objective, prescribing data were collected from electronic health records for 1 year following intervention discontinuation. We provided study results to surgeons after the 1-year follow-up concluded.

We estimated the persistent intervention effects at the discharged patient level using ordinary least squares regression for the same primary outcome. This procedure deviates from our original primary approach, as explained in the eMethods in Supplement 2. We estimated mean effects across the entire postintervention period and separately for each postintervention month. We controlled for preintervention outcome levels and clustered standard errors by randomization cluster. *P* < .05 was considered significant. Statistical analysis was performed using Stata/SE, version 18 (StataCorp LLC).

## Results

**Figure 1.**
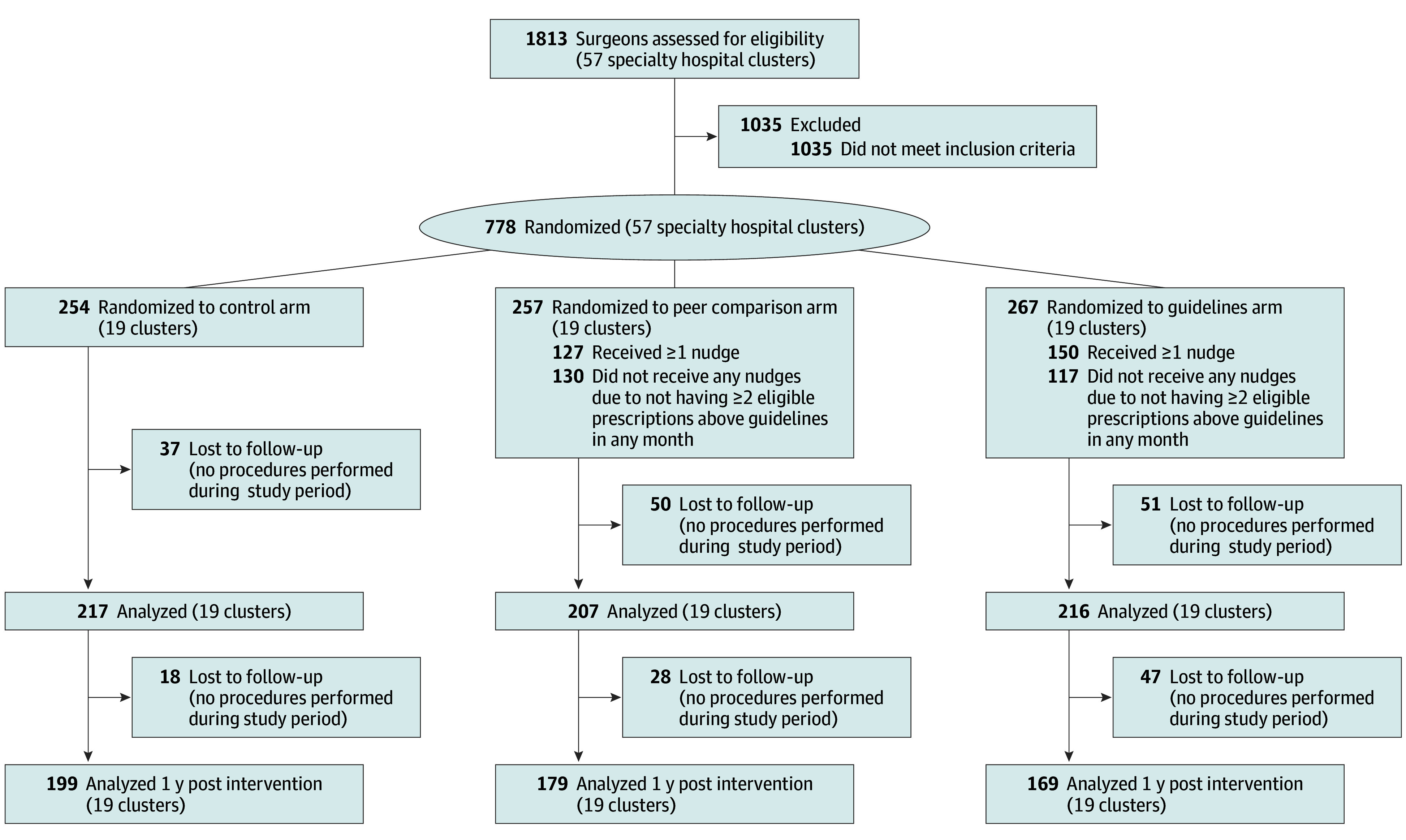
Flow Diagram

**Figure 2.**
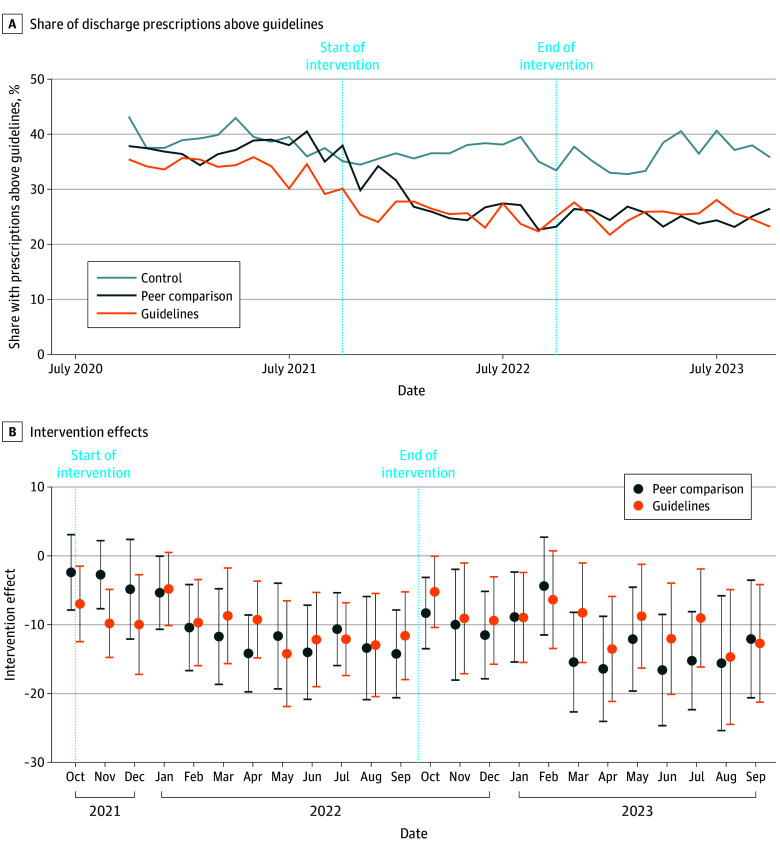
Prescribing Above Guideline Quantities Over Time by Intervention The intervention effects in each month, after controlling for baseline prescribing and with standard errors clustered by randomization cluster, are absolute percentage point differences. The error bars indicate 95% CIs.

In the preintervention period, among 778 surgeons randomized (245 in the control arm, 257 in the peer comparison arm, and 267 in the guidelines arm) (Figure 1), the share of discharges with an opioid prescription exceeding guideline recommendations was approximately 35% to 40% in each arm (Figure 2). During the intervention, the mean (SD) share was 25.5% (43.5%) in the guidelines arm, 27.5% (44.7%) for peer comparison, and 36.8% (48.2%) in the control arm. During the postintervention period, 34 024 patients were discharged after procedures performed by 547 surgeons (85% of 640 analyzed during the intervention period) (Figure 1).

Control surgeons prescribed above guidelines 36.3% of the time during the postintervention period vs 25.3% and 25.0% among guidelines and peer comparison surgeons, respectively (Figure 2). After adjusting for baseline above-guideline prescribing, peer comparison feedback significantly decreased guideline-discordant prescribing by 12.1 (95% CI, −18.8 to −5.5) percentage points (*P* = .001), and the guideline intervention significantly decreased guideline-discordant prescribing by 9.9 (95% CI, −17.9 to −1.9) percentage points (*P* = .02) in the postintervention period. The difference between intervention groups was not significant. Effect sizes were similar in each study month and remained significant 12 months post intervention.

## Discussion

This study shows that above-guideline prescribing of postsurgical opioids remained reduced 1 year after ceasing social norms–based feedback. Effect sizes in the postintervention period were nearly identical to those estimated with the same models during the intervention period,^3^ indicating sustained changes in prescribing behavior that remained integrated with surgery team practices. Reports of persistent change resulting from prescribing interventions are inconsistent; however, similar interventions using peer comparison feedback to improve guideline-concordant antibiotic prescribing have shown sustained postintervention improvements.^4,5^ Further research is needed to examine the mechanisms driving postintervention persistence. Possibilities include continuation of prescribing habits triggered by contextual clues, new prosocial identities as responsible prescribers, and ongoing discussions with peers reinforcing appropriate prescribing.^6^

Additional study of these feedback mechanisms could also optimize their implementation at scale. For example, shortening the intervention duration to 6 months may improve cost-effectiveness if the same persistent effect can be demonstrated. Our study is limited by only 1 year of postintervention follow-up. The sustained impact observed should continue to be evaluated over longer periods.

## Notes

Supplement 1.
Trial Protocol

Supplement 2. **eMethods.** Description of Statistical Approach
eReferences

Supplement 3.
Data Sharing Statement

## References

[1] Brummett CM, Waljee JF, Goesling J, . New persistent opioid use after minor and major surgical procedures in US adults. JAMA Surg. 2017;152(6):e170504. doi:10.1001/jamasurg.2017.0504 28403427 PMC7050825

[2] Schirle L, Stone AL, Morris MC, . Leftover opioids following adult surgical procedures: a systematic review and meta-analysis. Syst Rev. 2020;9(1):139. doi:10.1186/s13643-020-01393-8 32527307 PMC7291535

[3] Wagner Z, Kirkegaard A, Mariano LT, . Peer comparison or guideline-based feedback and postsurgery opioid prescriptions: a randomized clinical trial. JAMA Health Forum. 2024;5(3):e240077. doi:10.1001/jamahealthforum.2024.0077 38488780 PMC10943416

[4] Frost HM, Wittmer N, Keith A, Durfee MJ, Jenkins TC. Sustainability of interventions to increase guideline-concordant durations of antibiotic therapy for children with acute otitis media. J Pediatr. 2023;253:292-296.e2. doi:10.1016/j.jpeds.2022.09.004 36088995 PMC10231306

[5] Linder JA, Meeker D, Fox CR, . Effects of behavioral interventions on inappropriate antibiotic prescribing in primary care 12 months after stopping interventions. JAMA. 2017;318(14):1391-1392. doi:10.1001/jama.2017.11152 29049577 PMC5818848

[6] Frey E, Rogers T. Persistence: how treatment effects persist after interventions stop. Policy Insights Behav Brain Sci. 2014;1(1):172-179. doi:10.1177/2372732214550405

