# Supplementary material for: Persistence of Social Norms Feedback on Postsurgery Opioid Prescribing Behavior: Secondary Analysis of a Randomized Clinical Trial
Source: JAMA Health Forum. 2025 Jan 31;6(1):e245279. doi: 10.1001/jamahealthforum.2024.5279 (PMC11786227; doi:10.1001/jamahealthforum.2024.5279)
Supplement: Supplement 2. — eMethods. Description of Statistical Approach eReferences [file jamahealthforum-e245279-s002.pdf]

Supplemental Online Content

Zanocco KA, Wagner Z, Mariano LT, et al. Persistence of social norms feedback on postsurgery opioid prescribing behavior: secondary analysis of a randomized clinical trial. *JAMA Health Forum*. 2025;6(1):e245279. doi:10.1001/jamahealthforum.2024.5279

eMethods. Description of Statistical Approach

eReferences

This supplemental material has been provided by the authors to give readers additional information about their work.

© 2025 Zanocco KA et al. *JAMA Health Forum.*

eMethods. Description of Statistical Approach

In this paper, we used an ordinary least squares (OLS) regression model to estimate the effect of the interventions on guideline-discordant opioid prescribing after surgery. This modeling approach deviates from the primary modeling approach we used in the initial reporting of trial results in Wagner et al. (2024), where we used a hierarchical linear model (HLM) to estimate intervention impacts.[1] The HLM approach, which estimates a *cluster-level* average treatment effect, is not optimal for our main research questions, which pertain to the *participant-level* average treatment effect. The main effect of interest is whether the intervention reduced the likelihood that a patient received an opioid prescription above guidelines. The difference between the cluster-level average treatment effect and participant level average treatment effect are document in a recent paper by Kahan et al. that was published in 2023 (after our original analysis was pre-registered).[2] This paper shows that HLMs can be biased for the participant-level average treatment effect when there are unequal cluster sizes. In our case, the clusters are the hospital-specialty combinations, and the cluster sizes (the number of surgeries per cluster) range from 31 to 2,094. The HLM downweights higher-variance clusters, effectively downweighting the larger clusters and upweighting the smaller clusters, whereas the OLS approach weights each cluster according to the number of surgeries observed. In other words, OLS weights each surgery observed equally whereas HLMs give less importance to surgeries from high-volume clusters. Our interventions were most effective among surgeons who performed the highest volume of surgeries because these surgeons had more opportunities to see the email feedback (this is shown in figure 3a of Wagner et al. 2024).[1] Because the HLM gives the observations that were most impacted by the intervention less weight, this biases the estimate of the average effect for patients towards zero.

In preparing our original paper, we became aware of this issue and thus reported the OLS results in addition to the HLM results in eTable 6. The results from eTable 6 in Wagner et al. (2024) used the same exact model as our estimates from the current follow-up paper and thus are directly comparable. This comparison shows that effect sizes are quite similar in the follow-up period to the trial period. We chose to focus on the OLS modeling approach for the follow-up paper because of the issues described above, but for consistency we also estimated effects using the same HLM from the original paper, which included random effects for surgeon and specialty within hospital and control for baseline guideline-discordant opioid prescribing for each surgeon. Using the HLM, we found that the peer comparison feedback reduced guideline discordant prescribing by 3.9 percentage points (95% CI -9.8, 2.0; p=0.39) and the guideline discordant intervention reduced guideline discordant prescribing by 1.7 percentage points (95% CI -7.5, 4.2 ; p=0.57) in the postintervention period. For the reasons described above, these estimates are more reflective of the cluster-level effect and underestimate the effect for patients.

© 2025 Zanocco KA et al. *JAMA Health Forum.*

eReferences

1. Wagner Z, Kirkegaard A, Mariano LT, et al. Peer Comparison or Guideline-Based Feedback and Postsurgery Opioid Prescriptions: A Randomized Clinical Trial. JAMA Health Forum; 2024: American Medical Association; 2024. p. e240077-e.
2. Kahan BC, Li F, Copas AJ, Harhay MO. Estimands in cluster-randomized trials: choosing analyses that answer the right question. *International Journal of Epidemiology* 2023; **52**(1): 107-18.

© 2025 Zanocco KA et al. *JAMA Health Forum.*
