# Supplementary material for: Persistence of Social Norms Feedback on Postsurgery Opioid Prescribing Behavior: Secondary Analysis of a Randomized Clinical Trial
Source: JAMA Health Forum. 2025 Jan 31;6(1):e245279. doi: 10.1001/jamahealthforum.2024.5279 (PMC11786227; doi:10.1001/jamahealthforum.2024.5279)
Supplement: Supplement 3. — Data Sharing Statement [file jamahealthforum-e245279-s003.pdf]

Data Sharing Statement

Zanocco. Persistence of Social Norms Feedback on Postsurgery Opioid Prescribing Behavior. *JAMA Health Forum*. Published January 31, 2025. doi:10.1001/jamahealthforum.2024.5279

Data
Additional Information: ClinicalTrials.gov NCT05070338, https://classic.clinicaltrials.gov/ct2/show/NCT05070338
Data available: Yes
Data types: Deidentified participant data, Data dictionary
How to access data: Deidentified data will be made available on reasonable request to the principal investigator author(kwatkins@rand.org). Data aggregation of patents or providers may be used to prevent identifiability by inference.
When available: With publication

Supporting Documents
Document types: None

Additional Information
Who can access the data: researchers whose proposed use of the data has been approved
Types of analyses: for any purpose
Mechanisms of data availability: with a signed data access agreement
